# Energy Potential and Greenhouse Gas Emissions from Bioenergy Cropping Systems on Marginally Productive Cropland

**DOI:** 10.1371/journal.pone.0089501

**Published:** 2014-03-04

**Authors:** Marty R. Schmer, Kenneth P. Vogel, Gary E. Varvel, Ronald F. Follett, Robert B. Mitchell, Virginia L. Jin

**Affiliations:** 1 Agroecosystem Management Research Unit, United States Department of Agriculture-Agricultural Research Service (USDA-ARS), Lincoln, Nebraska, United States of America; 2 Grain, Forage and Bioenergy Research Unit, United States Department of Agriculture-Agricultural Research Service (USDA-ARS), Lincoln, Nebraska, United States of America; 3 Soil-Plant Nutrient Research Unit, United States Department of Agriculture-Agricultural Research Service (USDA-ARS), Ft. Collins, Colorado, United States of America; North Carolina State University, United States of America

## Abstract

Low-carbon biofuel sources are being developed and evaluated in the United States and Europe to partially offset petroleum transport fuels. Current and potential biofuel production systems were evaluated from a long-term continuous no-tillage corn (*Zea mays* L.) and switchgrass (*Panicum virgatum* L.) field trial under differing harvest strategies and nitrogen (N) fertilizer intensities to determine overall environmental sustainability. Corn and switchgrass grown for bioenergy resulted in near-term net greenhouse gas (GHG) reductions of −29 to −396 grams of CO_2_ equivalent emissions per megajoule of ethanol per year as a result of direct soil carbon sequestration and from the adoption of integrated biofuel conversion pathways. Management practices in switchgrass and corn resulted in large variation in petroleum offset potential. Switchgrass, using best management practices produced 3919±117 liters of ethanol per hectare and had 74±2.2 gigajoules of petroleum offsets per hectare which was similar to intensified corn systems (grain and 50% residue harvest under optimal N rates). Co-locating and integrating cellulosic biorefineries with existing dry mill corn grain ethanol facilities improved net energy yields (GJ ha^−1^) of corn grain ethanol by >70%. A multi-feedstock, landscape approach coupled with an integrated biorefinery would be a viable option to meet growing renewable transportation fuel demands while improving the energy efficiency of first generation biofuels.

## Introduction

Reduction in greenhouse gas (GHG) emissions from transportation fuels can result in near- and long-term climate benefits [1]. Biofuels are seen as a near-term solution to reduce GHG emissions, reduce U.S. petroleum import requirements, and diversify rural economies. Depending on feedstock source and management practices, greater reliance on biofuels may improve or worsen long-term sustainability of arable land. U.S. farmers have increased corn (*Zea mays* L.) production to meet growing biofuel demand through land expansion, improved management and genetics, increased corn plantings, or by increased continuous corn monocultures [2]–[4]. Productive cropland is finite, and corn expansion on marginally-productive cropland may lead to increased land degradation, including losses in biodiversity and other desirable ecosystem functions [4]–[6]. We define marginal cropland as fields whose crop yields are 25% below the regional average. The use of improved corn hybrids and management practices have increased U.S. grain yields by 50% since the early 1980's [7] with an equivalent increase in non-grain biomass or stover yields. Corn stover availability and expected low feedstock costs make it a likely source for cellulosic biofuel. However, excessive corn stover removal can lead to increased soil erosion and decreased soil organic carbon (SOC) [8] which can negatively affect future grain yields and sustainability. Biofuels from cellulosic feedstocks (e.g. corn stover, dedicated perennial energy grasses) are expected to have lower GHG emissions than conventional gasoline or corn grain ethanol [9]–[13]. Furthermore, dedicated perennial bioenergy crop systems such as switchgrass (*Panicum virgatum* L.) have the ability to significantly increase SOC [14]–[16] while providing substantial biomass quantities for conversion into biofuels under proper management [17], [18].

Long-term evaluations of feedstock production systems and management practices are needed to validate current and projected GHG emissions and energy efficiencies from the transportation sector. In a replicated, multi-year field study located 50 km west of Omaha, NE, we evaluated the potential to produce ethanol on marginal cropland from continuously-grown no-tillage corn with or without corn residue removal (50% stover removal) and from switchgrass harvested at flowering (August) versus a post-killing frost harvest. Our objectives were to compare the effects of long-term management practices including harvest strategies and N fertilizer input intensity on continuous corn grain and switchgrass to determine ethanol production, potential petroleum offsets, and net energy yields. We also present measured SOC changes (0 to 1.5 m) over a nine year period from our biofuel cropping systems to determine how direct SOC changes impact net GHG emissions from biofuels. Furthermore, we evaluate the potential efficiency advantages of co-locating and integrating cellulosic conversion capacity with existing dry mill corn grain ethanol plants.

## Materials and Methods

This study is located on the University of Nebraska Agricultural Research and Development Center, Ithaca, Nebraska, USA on a marginal cropland field with Yutan silty clay loam (fine-silty, mixed, superactive, mesic Mollic Hapludalf) and a Tomek silt loam (fine, smectitic, mesic Pachic Argiudoll) soil. Switchgrass plots were established in 1998 and continuous corn plots were initiated in 1999. The study is a randomized complete block design (replications = 3) with split-split plot treatments. Main treatments are two cultivars of switchgrass, ‘Trailblazer’ and ‘Cave-in-Rock’, and a glyphosate tolerant corn hybrid. Main treatment plots are 0.3 ha which enables the use of commercial farm equipment. Switchgrass is managed as a bioenergy crop, and corn is managed under no-tillage conditions (no-till farming since 1999). Split-plot treatments are nitrogen (N) fertilizer levels and split-split plots are harvest treatments. Annual N fertilizer rates (2000–2007) were 0 kg N ha^−1^, 60 kg N ha^−1^, 120 kg N ha^−1^, and 180 kg N ha^−1^ as NH_4_NO_3_, broadcast on the plots at the start of the growing season. The 0 kg N ha^−1^, 60 kg N ha^−1^, 120 kg N ha^−1^ fertilizer rates were used on switchgrass [19] while the 60 kg N ha^−1^, 120 kg N ha^−1^, and 180 kg N ha^−1^ fertilizer rates were used for corn. Switchgrass harvest treatments were initiated in 2000 and consist of a one-cut harvest either in early August or after a killing frost. Corn stover treatments were initiated in 2000 and are either no stover harvest or stover removal, where the amount of stover removed approximates 50% of the aboveground biomass after corn grain is harvested.

Baseline soil samples were taken in 1998 at the center of each subplot and re-sampled in 2007 at increments of 0–5, 5–10, 10–30, 30–60, 60–90, 90–120, and 120–150 cm depths [15]. Average changes in total SOC (0–1.5 m) from 1998–2007 were used to estimate direct soil C changes. Further management practices and detailed soil property values from this study have been previously reported [15], [20]. Summary of petroleum offsets (GJ ha^−1^), ethanol production (L ha^−1^), greenhouse gas (GHG) emissions (g CO_2_e MJ^−1^), net GHG emissions (Mg CO_2_e ha^−1^), and GHG reductions (%) for corn grain, corn grain with stover removal, and switchgrass are presented in Table S1 in File S1.

### Statistical Analyses

Yield data analyzed were from 2000 to 2007, where 2000 was the initiation of harvest treatments for continuous corn and switchgrass and 2007 was the last year that SOC was measured for this study. Data from switchgrass cultivars were pooled together based on their similar aboveground biomass yields over years and similar changes in SOC [15]. Data were analyzed using a linear mixed model approach with replications considered a random effect. Mean separation tests were conducted using the Tukey-Kramer method. Significance was set at P≤0.05.

### Life-cycle assessment

For energy requirements in the production, conversion, and distribution of corn grain ethanol and cellulosic ethanol, values from the Greenhouse Gases, Regulated Emissions, and Energy Use in Transportation (GREET v. 1.8) [21], Energy and Resources Group Biofuel Analysis Meta-Model (EBAMM) [22], and Biofuel Energy Systems Simulator (BESS) [23] life cycle assessment models were used as well as previous agricultural energy estimates for switchgrass [12]. Energy use in the agricultural phase consisted of agricultural inputs (seed, herbicides, fertilizers, packaging), machinery energy use requirements, material transport, and diesel requirements used in this study. Stover energy requirements from the production phase were from the diesel requirements to bale, load, and stack corn stover and the embodied energy of the farm machinery used. A proportion of the N fertilizer and herbicide requirements were allocated to the amount of stover harvested.

Multiple biorefinery configurations are presented to evaluate different conversion scenarios and how this affects GHG emissions, petroleum offset credits, and net energy yield (NEY) values. Biorefinery scenarios evaluated in this study are: (i) a natural gas (NG) dry mill corn grain ethanol plant with dry distillers grain (DDGS) as a co-product for the corn grain-only harvests [23]–[25], (ii) a co-located dry mill corn grain and cellulosic ethanol plant with combined heat and power (CHP) and DDGS co-product, where corn stover is primarily used to displace dry mill ethanol plant natural gas requirements [25], [26], (iii) and a standalone cellulosic (switchgrass or corn stover) ethanol plant (sequential hydrolysis and fermentation) with CHP capability and electricity export [22], [27]–[29]. Chemical and enzyme production costs and related GHG emissions for corn grain and cellulosic conversion to ethanol were also incorporated [28]. Ethanol recovery for corn grain was estimated to be 0.419 L kg^−1^ [23]. Ethanol recovery for corn stover and switchgrass were based on cell wall composition from harvested biomass samples. Ground aboveground switchgrass samples were scanned using a near-infrared spectrometer to predict cell wall and soluble carbohydrate biomass composition [30]. Ground corn stover samples were analyzed using a near-infrared spectrometer-based calibration equation developed by the National Renewable Energy Laboratory to predict corn stover cell wall composition [31]. Switchgrass and corn stover cell wall conversion to ethanol was based on composition components of glucan, xylose and arabinose [30], [31]. Glucan to ethanol conversion was assumed to be 85.5%, and xylose and arabinose was estimated to have 85% ethanol recovery efficiency [29]. Estimated ethanol recovery for corn stover was 327 L Mg^−1^ which was similar to other findings [29]. For switchgrass, ethanol recovery based on glucan, xylose, and arabinose concentrations was estimated to be 311 L Mg^−1^ and 344 L Mg^−1^ for an August harvest and a post-frost harvest, respectively.

Ethanol plant size capacity was estimated to be 189 million L yr^−1^ for the corn grain-only and cellulosic-only scenarios. For the co-located facility, total plant size was assumed to be 378 million L yr^−1^ capacity. Fossil fuel energy requirements for the conventional corn grain ethanol plant is assumed to be 7.69 MJ L^−1^ for natural gas to power the plant and to dry DGS, 0.59 MJ L^−1^ for corn grain transportation from farm to ethanol plant, 0.67 MJ L^−1^ for electricity purposes, 0.13 MJ L^−1^ to capital depreciation costs, and 0.58 MJ L^−1^ for wastewater processing and effluent restoration [10], [22]. Fossil fuel requirements for the corn grain/cellulosic ethanol plant are feedstock transportation 0.63 MJ L^−1^ for corn stover, 0.59 MJ L^−1^ for corn grain transportation from farm to ethanol plant, 0.44 MJ L^−1^ to capital depreciation costs, and 0.58 MJ L^−1^ for wastewater treatment and processing (Table S2 in File S1). Cellulosic ethanol plant fossil fuel requirements are 0.63 MJ L^−1^ for switchgrass transportation from field to ethanol plant, 0.06 MJ L^−1^ diesel requirements for biomass transport within the ethanol plant grounds, 0.44 MJ L^−1^ to capital depreciation costs, and 0.58 MJ L^−1^ for wastewater processing, effluent restoration, and recovery (Table S2 in File S1).

For the co-located corn grain and cellulosic facility, we assumed (i) power and electrical utilities were shared [26]; (ii) power requirements were supplied mainly from the lignin portion of stover with combined ethanol purification from the starch and cellulosic ethanol conversion pathways [26]; and (iii) extra stover biomass would be required in addition to the lignin to meet steam requirements. A co-location facility would require additional unprocessed bales to be used in addition to lignin which lowered the amount of ethanol being generated from stover at a co-located facility compared to a standalone cellulosic facility that uses stover as their primary feedstock (Table S1 in File S1). Electricity would be imported from the grid in this scenario and DDGS exported as the only co-product. Recent analysis [29] of converting cellulose to ethanol has estimated a higher internal electrical demand than previously assumed [26]; suggesting electricity export under this configuration would be unlikely. The value of DDGS as animal feed would likely preclude its use in meeting power requirement in a co-located facility. We based our total biomass energy requirement on the lignin concentration in stover and the expected biomass energy use requirements to power a co-located ethanol plant [25]. Estimated biomass requirements were 11 MJ L^−1^ ethanol and embodied energy value of 16.5 MJ kg^−1^ (low heating value) for stover biomass.

Net energy yield (NEY) values (renewable output energy – fossil fuel input energy) were calculated for each feedstock and conversion scenario. Output energy was calculated from ethanol output plus co-product credits. Co-product credit for DDGS is 4.13 MJ L^−1^ for the corn grain-only ethanol plant and the co-located corn grain/cellulosic ethanol plant [32]. Electricity co-product credit for standalone cellulosic ethanol was estimated to be 1.68 MJ L^−1^ [29]. Petroleum offsets (GJ ha^−1^) were calculated in a similar fashion as NEY with total ethanol production (MJ ha^−1^) along with petroleum displacement from co-products minus petroleum inputs consumed in the production, conversion, and distribution phase (Tables S1 and S3 in File S1). Petroleum offsets were calculated as the difference between ethanol output and petroleum inputs from the agricultural, conversion, and distribution phase (Table S1 in File S1). Petroleum requirements for each cropping system were calculated from input requirements from this study and derived values from the EBAMM model [22]. For input requirements without defined petroleum usage, we used the default parameter in EBAMM that estimates U.S. average petroleum consumption at 40% for input source. Petroleum offset credits associated with corn grain ethanol co-products were estimated to be 0.71 MJ L^−1^ while credits for corn stover and switchgrass cellulosic ethanol co-products (standalone facility) were 0.12 MJ L^−1^ (Table S3 in File S1). Petroleum offset credits were calculated from GREET (v 1.8).

### Greenhouse gas emissions

Greenhouse gas offsets associated with the production of corn grain and cellulosic ethanol were modeled from the EBAMM and BESS models [22], [23]. Agricultural GHG emissions were based on fuel use, fertilizer use, herbicide use, farm machinery requirements, and changes in SOC. Direct land use change by treatment plot can either be a GHG source or a GHG sink depending on SOC changes from this study [15]. Co-product GHG credits for DDGS or electricity export were derived from the BESS [23] and GREET (v. 1.8) models [21]. Co-product GHG credits for DDGS was −347 g CO_2_e L^−1^ ethanol and −304 g CO_2_e L^−1^ ethanol for cellulosic electricity export (Table S4 in File S1). Indirect land use changes for corn grain ethanol or switchgrass were not estimated in this analysis. GHG offsets were calculated on both an energy and areal basis (Table S1 in File S1).

Greenhouse gas emissions from N fertilizer were evaluated from the embodied energy requirements and subsequent nitrous oxide (N_2_O) emissions (Table S4 in File S1). Direct and indirect nitrous oxide emissions were calculated in this study using Tier 1 Intergovernmental Panel on Climate Change calculations. Greenhouse gas emission values for the agricultural phase are included in Table S4 in File S1 and for the conversion and distribution phase in Table S5 in File S1. For the agricultural phase, total GHG emissions were calculated from the production of fertilizers, herbicides, diesel requirements, drying costs for corn grain, and the embodied energy in farm machinery minus direct soil C changes occurring for the study period (Table S4 in File S1). GHG emissions were reported on an energy basis, areal basis, and the difference between ethanol and conventional gasoline (Table S1 in File S1). For net GHG emissions (Mg CO_2_e ha^−1^), calculations were based on GHG intensity values (g CO_2_e MJ^−1^) multiplied by biofuel production (MJ ha^−1^) for each cropping system. GHG reductions (Table S1 in File S1) were calculated as the percent difference from conventional gasoline as reported by the California Air Resource Board (99.1 g CO_2_ MJ^−1^) [33].

## Results and Discussion

**Figure 1:**
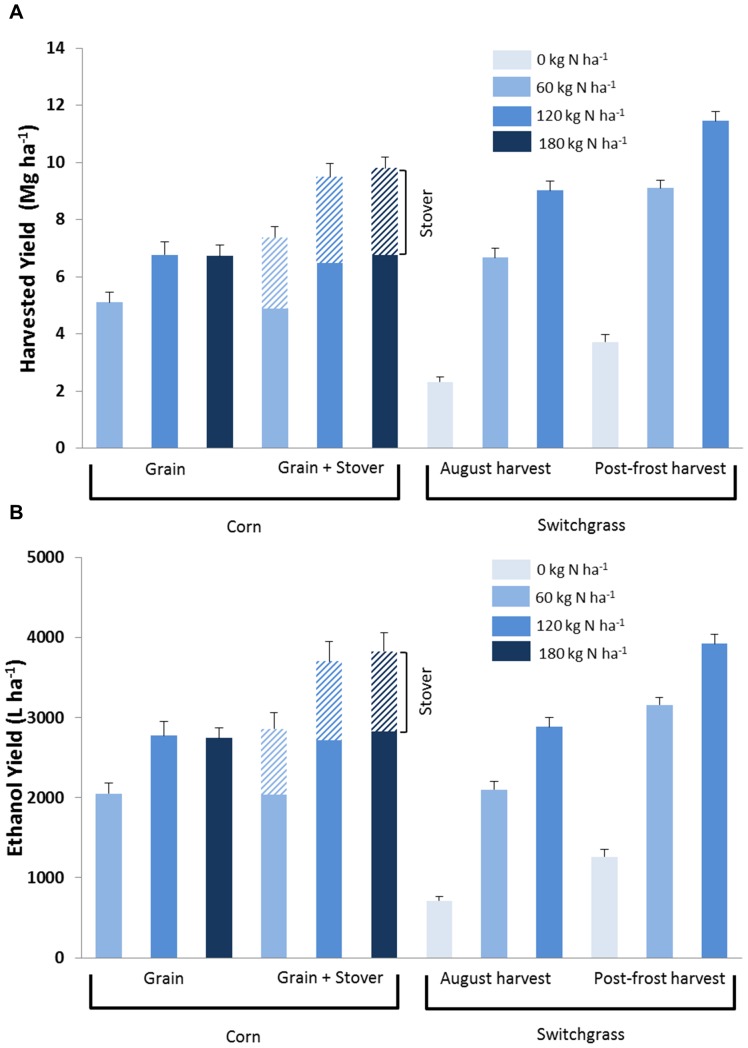
Harvested mean annual yield ± standard error (A) and ethanol energy ± SEM (B) for no-till continuous corn (grain-only harvest or grain and stover harvest) and switchgrass (August harvest or Post-frost harvest) under variable nitrogen rates on marginally-productive rainfed cropland for 2000–2007 (n = 3 replicate corn system plots and 6 replicate switchgrass plots).

Harvest and N fertilizer management treatments affected grain and biomass yields in both crops over eight growing seasons (Fig. 1A). Switchgrass harvested after a killing frost had 27% to 60% greater biomass yields compared with an August harvest under similar fertilization rates. Highest harvested biomass yields (mean = 11.5 Mg ha^−1^ yr^−1^) were from fertilized (120 kg N ha^−1^) switchgrass harvested after a killing frost while continuous corn showed similar grain and stover yields [factorial analysis of variance (ANOVA), P = 0.72] under the highest N fertilizer levels (180 kg N ha^−1^) (Fig. 1A).

Potential ethanol yields varied from 2050 to 2774 L ha^−1^ yr^−1^ for corn grain-only harvests while those for corn grain with stover removal ranged from 2862 to 3826 L ethanol ha^−1^ yr^−1^ (Fig. 1B). Ethanol contribution from corn stover ranged from 820 to 998 L ha^−1^ yr^−1^ when stover is converted at a standalone cellulosic plant (Fig. 1B). Separate ethanol facilities showed slightly higher potential ethanol yields (L ha^−1^) than at a co-located facility (Table S1 in File S1) because a larger portion of corn stover biomass was required to meet thermal power requirements at a co-located facility (SI text in File S1). Unfertilized switchgrass had potential ethanol yield values similar to corn stover. Switchgrass under optimal management practices had 17% higher biomass yields than the highest yielding corn with stover removal treatment. Potential ethanol yield for switchgrass, however, was similar (factorial ANOVA, P>0.05) to corn with stover removal (Fig. 1B) due to lower cellulosic ethanol recovery efficiency than exists for corn grain ethanol conversion efficiency. Switchgrass ethanol conversion efficiency from this study was based on updated biochemical conversion processes [29] using known cell wall characteristics [30] that result in lower conversion rates than previous estimates [12], [18].

**Figure 2:**
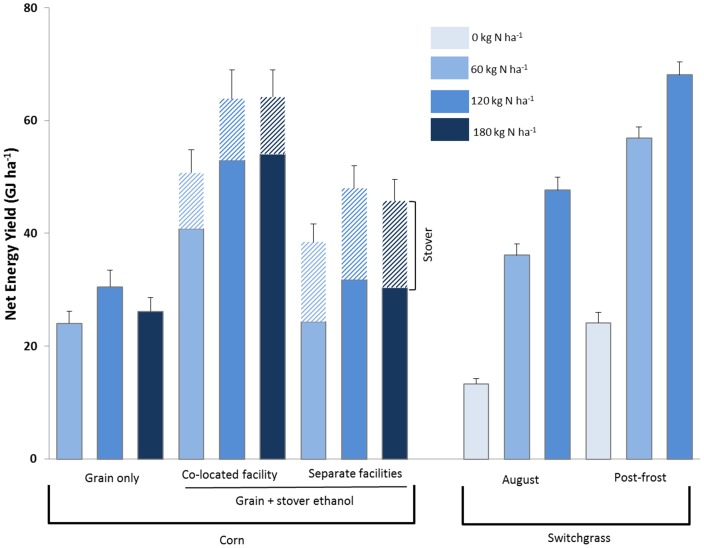
Net energy yield ± standard error for no-till continuous corn (grain-only or grain and stover harvest) and switchgrass (August harvest or post-frost harvest) under variable nitrogen rates on marginally-productive cropland (n = 3 replicate corn system plots and 6 replicate switchgrass plots). Conversion processes evaluated include corn grain-only harvest at a natural gas (NG) dry mill, corn grain with stover harvest at a co-located facility (lignin portion of stover used as primary energy source for grain and cellulose conversion), corn grain with stover harvest at separate ethanol facilities (NG dry mill and a cellulosic ethanol plant), and switchgrass (cellulosic ethanol plant).

Net energy yield (NEY) (renewable output energy minus fossil fuel input energy) and GHG emission intensity (grams of CO_2_ equivalents per megajoule of fuel, or g CO_2_e MJ^−1^) are considered the two most important metrics in estimating fossil fuel replacement and GHG mitigation for biofuels [34]. Switchgrass harvested after a killing frost (120 kg N ha^−1^) and the co-located grain and stover conversion pathway (120 kg N ha^−1^ and 180 kg N ha^−1^ treatments) had the highest overall NEY values (Fig. 2). Net energy yields for continuous corn were higher at a co-located facility because stover biomass and lignin replaced natural gas for thermal energy (Fig. 2). Ethanol conversion of corn grain and stover at separate facilities was intermediate in NEY while traditional corn grain-only natural gas (NG) dry mill ethanol plants had the lowest NEY values for the continuous corn systems. Delaying switchgrass harvest from late summer to after a killing frost resulted in significant improvement in NEY and potential ethanol output under similar N rates. Unfertilized switchgrass had similar NEY values compared with corn grain processed at a NG dry mill ethanol plant (factorial ANOVA, P = 0.12) while fertilized switchgrass harvested after a killing frost had higher NEY values (factorial ANOVA, P<0.0001) than NG dry mill corn grain ethanol plants (Fig. 2).

**Figure 3:**
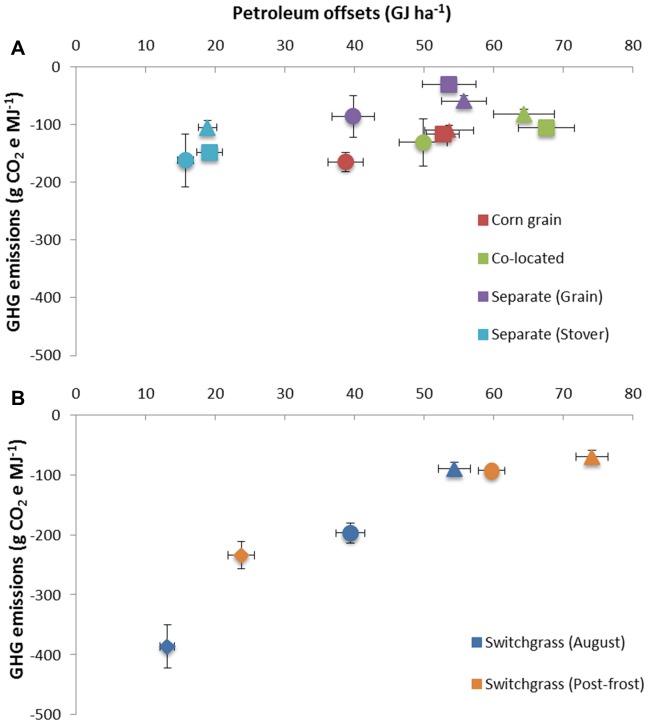
Petroleum offsets compared with GHG emissions (g CO_2_e MJ^−1^ ethanol) for continuous corn and switchgrass grown on marginally-productive cropland (n = 3 replicate corn system plots and 6 replicate switchgrass plots). (A) Continuous corn values represent harvest method (stover harvested or retained) and ethanol conversion pathway (co-located facility or at a separate ethanol facilities). (B) Switchgrass values are based on harvest date and N fertilizer rate. Fertilizer rates are 0 kg N ha^−1^ (♦), 60 kg N ha^−1^ (•), 120 kg N ha^−1^ (▴), and 180 kg N ha^−1^ (▪). Error bars indicate standard errors of the mean.

Both the continuous corn and switchgrass systems showed significant petroleum offset (ethanol output minus petroleum inputs) capability, with the intensified bioenergy cropping systems having the highest petroleum offsets (Fig. 3). Petroleum use varied by cropping system in the agricultural phase with continuous corn systems having higher overall petroleum requirements than switchgrass. Petroleum requirements (mainly diesel fuel) to harvest corn stover are small relative to corn grain harvest as a result of low harvested stover yields. Lowest petroleum offsets for continuous corn systems were from stover harvests at a separate dedicated cellulosic facility (Table S1 in File S1). Corn grain-only harvests offset less petroleum compared with grain and stover at separate ethanol facilities under similar fertilizer rates (factorial ANOVA, P<0.01). Management practices in switchgrass resulted in the largest variation in petroleum offset credits (Fig. 3B). Petroleum offsets (GJ ha^−1^) were positively associated with NEY values [−1.81+0.84 (Petroleum offset); (P<0.0001); (R^2^ = 0.76)], indicating that bioenergy cropping systems with large NEY values will likely result in higher petroleum displacement.

All bioenergy cropping systems evaluated in our study had SOC sequestration rates exceeding 7.3 Mg CO_2_ yr^−1^ (Table S4 in File S1), with over 50% of SOC sequestration occurring below the 0.3 m soil depth [15]. Soil organic C increased even with corn stover removal, indicating that removal rates were sustainable in terms of SOC and grain yield for this time period. No-tillage continuous corn systems have lower stover retention requirements to maintain SOC than continuous corn with tillage or corn-soybean (*Glycine max* (L.) Merr.) rotations [8]. Consequently, all conversion pathways had negative GHG emission values as a result of SOC sequestration offsetting GHG emissions from the production, harvest, conversion and distribution phases for corn grain ethanol and cellulosic ethanol. For switchgrass, SOC storage values were similar to other findings within the same ecoregion [16] and a long-term Conservation Reserve Program grassland [35]. Measured SOC storage from the continuous corn systems (Table S4 in File S1) were significantly higher than modeled SOC storage estimates from this region [36]. Corn grain grown with low N rates (60 kg ha^−1^) had GHG intensity values similar to continuous corn under optimum N rates (120 kg ha^−1^) but resulted in lower ethanol yields and lower petroleum offset potential (Fig. 3A). Lowest GHG emission intensity values on an energy basis (g CO_2_e MJ^−1^) were from unfertilized switchgrass (Table S1 in File S1) due to lower ethanol yields, lower agricultural energy emissions, and similar SOC storage compared with the other biofuel cropping systems. For switchgrass, management practices that resulted in the lowest GHG emission on an energy basis resulted in the lowest petroleum offset potential (Fig. 3B). Direct N_2_O emissions (Table S4 in File S1) were estimated using Intergovernmental Panel on Climate Change methodology and are in agreement with study site N_2_O flux measurements from a later time series which indicated N rate as the major contributor to N_2_O emissions [37]. When evaluating GHG emissions on a per unit area basis (g CO_2_e ha^−1^), unfertilized switchgrass and corn grain-only systems showed similar results with the more intensified cropping systems (Table S1 in File S1).

Both switchgrass and continuous corn with stover removal produced similar ethanol potential, NEY values, petroleum offsets, and GHG emissions but overall values and metric efficiencies were dependent on management practices and downstream conversion scenarios. Dedicated perennial grass systems used for bioenergy will need to have similar or greater yield potential than existing annual crops for widespread adoption to meet renewable energy demands and provide similar economic returns to producers. We have previously shown that switchgrass ethanol yields were comparable with regional corn grain ethanol yields [12]. Here we demonstrate that when switchgrass is optimally managed, ethanol potential is similar to a continuous corn cropping system with stover removal and exceeds ethanol yield for corn grain-only systems on marginally-productive cropland. Furthermore, breeding improvements for bioenergy specific switchgrass cultivars have shown higher yield potential than cultivars evaluated here [38].

Coupling sustainable agricultural residue harvests with dedicated energy crops improves land-use efficiency and reduces biomass constraints for a mature cellulosic biofuel industry. Recent analysis has shown that sufficient land exists in the U.S. Corn Belt to support a cellulosic ethanol industry without impacting productive cropland [18], [39], [40]. The effect of dedicated energy crops and corn grain on indirect land use change varies significantly based on the assumptions and models used [13], [41], [42] but bioenergy crops grown on marginally-productive cropland will have less impact on indirect land use change than bioenergy crops grown on more productive cropland. Likewise, model assumptions underlying direct SOC sequestration will impact system evaluations of GHG emissions and mitigation. Measured SOC sequestration values presented here were based on production years evaluated and were not extrapolated beyond this time-frame. Extrapolating SOC values from this time-frame to a 30-yr time horizon or 100-yr time horizon is still larger than current life cycle assessment assumptions on SOC sequestration potential of switchgrass or no-till corn [12], [42], [43]. This highlights the importance of accounting for direct SOC changes at depth to accurately estimate GHG emissions for biofuels under both marginal and productive cropland. Further long term evaluation of management practices (e.g. tillage, stover removal) on SOC sequestration potential for corn grain systems under irrigated conditions on productive cropland is warranted [44].

A multi-feedstock, landscape approach minimizes economic and environmental risks in meeting feedstock demands for cellulosic ethanol production by providing sufficient feedstock availability while maintaining ecosystem services. A co-located cellulosic biorefinery is expected to have economic advantages by reducing capital costs requirements for cellulosic conversion and through sharing of infrastructure costs. In this study, we used corn stover as the feedstock for the co-located cellulosic biorefinery but the benefits will apply to other cellulosic feedstocks. A co-located facility can increase NEY values by decreasing natural gas use for thermal energy, but current and forecasted U.S. natural gas prices [45] may affect large scale adoption of co-location unless there are incentives for displacing fossil energy in existing NG dry mill ethanol plants [46]. Integrating cellulosic refining capacity with existing corn grain ethanol plants can improve the sustainability of first generation biofuels and enable the implementation of cellulosic biofuels into the U.S. transportation sector.

## Supporting Information

File S1 **Tables S1–S5.**
(DOCX)
