# Supplementary material for: Energy Potential and Greenhouse Gas Emissions from Bioenergy Cropping Systems on Marginally Productive Cropland
Source: PLoS One. 2014 Mar 4;9(3):e89501. doi: 10.1371/journal.pone.0089501 (PMC3942357; doi:10.1371/journal.pone.0089501)
Supplement: File S1 — Tables S1–S5. (DOCX) [file pone.0089501.s001.docx]

**Supporting information for**

**Energy potential and greenhouse gas emissions from bioenergy cropping systems on marginally productive cropland**

**M.R. Schmer, K.P. Vogel, G.E. Varvel, R.F. Follett, R.B. Mitchell, V.L. Jin**

Table S1. Ethanol production, petroleum offset, greenhouse gas (GHG) emissions, net GHG emissions, and GHG reduction for corn grain, corn grain with stover removal, and switchgrass (August and post-frost harvest) grown on marginally-productive cropland (mean ± standard error) .

| Cropping system | Conversion  Process* | N Rate | Ethanol  Yield | | | Petroleum  Offset** | | | GHG  emission | | | Net GHG | | | GHG  reduction^†^ |
| --- | --- | --- | --- | --- | --- | --- | --- | --- | --- | --- | --- | --- | --- | --- | --- |
|  |  | ─kg ha^-1^─ | ─L ha^-1^─ | | | ─GJ ha^-1^─ | | | ─g CO_2_e MJ^-1^─ | | | ─Mg CO_2_e ha^-1^─ | | | ──%── |
| Corn grain | NG dry mill | 60 | 2050 | ± | 131 | 39 | ± | 2.6 | -167 | ± | 17 | -6.9 | ± | 0.7 | 266 |
|  |  | 120 | 2774 | ± | 181 | 54 | ± | 3.7 | -108 | ± | 9 | -5.9 | ± | 0.3 | 207 |
|  |  | 180 | 2744 | ± | 123 | 53 | ± | 2.4 | -117 | ± | 11 | -6.6 | ± | 0.6 | 216 |
|  |  |  |  |  |  |  |  |  |  |  |  |  |  |  |  |
| Corn grain and stover | Co-located | 60 | 2676 | ± | 183 | 50 | ± | 3.6 | -130 | ± | 41 | -6.5 | ± | 2.1 | 230 |
|  |  | 120 | 3413 | ± | 230 | 64 | ± | 4.6 | -78 | ± | 9 | -5.3 | ± | 0.5 | 177 |
|  |  | 180 | 3568 | ± | 215 | 68 | ± | 4.2 | -104 | ± | 10 | -7.4 | ± | 0.6 | 203 |
|  |  |  |  | | |  |  |  |  |  |  |  |  |  |  |
| Corn grain and stover | Separate | 60 | 2042(820 | ±± | 197  61) | 40  (16 | ±  ± | 3.2  1.2) | -85  (-161 | ±  ± | 36  46) | -3.2  (-2.4 | ±  ± | 1.4  0.7) | 185  (260) |
|  |  | 120 | 2719(984 | ±± | 248  70) | 54  (19 | ±± | 4.1  1.4) | -29  (-101 | ±± | 7  12) | -1.3  (-1.9 | ±± | 0.2  0.2) | 128  (201) |
|  |  | 180 | 2828(998 | ±± | 230  95) | 56  (19 | ±  ± | 3.4  1.8) | -58  (-146 | ±  ± | 9  13) | -3.3  (-2.7 | ±  ± | 0.5  0.1) | 157  (246) |
|  |  |  |  | | |  |  |  |  |  |  |  |  |  |  |
| Switchgrass | Cellulosic | 0 | 712 | ± | 53 | 13 | ± | 1.0 | -396 | ± | 36 | -4.8 | ± | 0.3 | 495 |
| (August) |  | 60 | 2099 | ± | 105 | 40 | ± | 2.0 | -202 | ± | 26 | -8.0 | ± | 0.6 | 301 |
|  |  | 120 | 2881 | ± | 116 | 54 | ± | 2.2 | -91 | ± | 10 | -5.5 | ± | 0.5 | 190 |
|  |  |  |  | | |  |  |  |  |  |  |  |  |  |  |
| Switchgrass | Cellulosic | 0 | 1263 | ± | 95 | 24 | ± | 1.8 | -236 | ± | 23 | -5.2 | ± | 0.5 | 336 |
| (Post-frost) |  | 60 | 3155 | ± | 97 | 60 | ± | 1.9 | -92 | ± | 9 | -5.9 | ± | 0.6 | 192 |
|  |  | 120 | 3919 | ± | 117 | 74 | ± | 2.2 | -68 | ± | 11 | -5.3 | ± | 0.9 | 167 |

*Conversion processes evaluated include corn grain-only harvest at a natural gas (NG) dry mill, corn grain with stover harvest at a co-located facility (lignin portion of stover used as primary energy source for grain and cellulose conversion), corn grain with stover harvest at separate ethanol facilities (NG dry mill and cellulosic ethanol plant), and switchgrass (cellulosic ethanol plant). Values in parentheses represent corn stover.

**For continuous corn, petroleum use averaged 3210 MJ ha^-1^, 3288 MJ ha^-1^, and 3365 MJ ha^-1^ for the 60 kg N ha^-1^, 120 kg N ha^-1^, and 180 kg N ha^-1^ treatments, respectively. For continuous corn with stover removal, petroleum use averaged 3551 MJ ha^-1^, 3676 MJ ha^-1^, and 3751 MJ ha^-1^ for the 60 kg N ha^-1^, 120 kg N ha^-1^, and 180 kg N ha^-1^ treatments, respectively. Petroleum requirements for switchgrass was 764 MJ ha^-1^, 1584 MJ ha^-1^, and 1904 MJ ha^-1^ for the 0 kg N ha^-1^, 60 kg N ha^-1^, and 120 kg N ha^-1^ treatments, respectively.

†GHG reductions are given as the percent difference relative to conventional gasoline (99.1 g CO_2_ MJ^-1^) for each crop type and conversion pathway.

Table S2. Agriculture and biorefinery energy inputs and energy values for corn grain, corn stover, and switchgrass. Values in parenthesis are for corn stover.

|  |  |  |  | Co-located |  |
| --- | --- | --- | --- | --- | --- |
| Production Inputs | Switchgrass | Corn Grain | Stover | Grain (Stover) | Source(s) |
| VARIABLE INPUTS | ────────────MJ kg^-1^──────────── | | | |  |
| Nitrogen Fertilizer* | 66.0 | 66.0 | 66.0 | 66.0 | (1) |
| Material Transport | 0.65 | 0.65 | 0.65 | 0.65 | (2) |
| Packaging | 0.27 | 0.27 | 0.27 | 0.27 | (2) |
|  |  |  |  |  |  |
| FIXED INPUTS | ────────────MJ ha^-1^──────────── | | | |  |
| Seed | 0 | 181 | 0 | 181 | (3) |
| Herbicide | 85** | 1330 | *** | 1330 | (1) |
| LPG (grain drying) | 0 | 765 | 0 | 765 | (4, 5) |
| Farm machinery† | 325 | 410 | 172 | 410 (172) | (1) |
| Farm diesel use | ‡ | 1521 | 313 | 1521 (313) | (5) |
|  |  |  |  |  |  |
| CONVERSION INPUTS | ────────────MJ L^-1^──────────── | | | |  |
| Feedstock transport | 0.63 | 0.59 | 0.63 | 0.59 (0.63) | (2) |
| Natural Gas | 0 | 7.69 | 0 | 0 | (5) |
| Electricity | 0 | 0.67 | 0 | 0.67 | (2) |
| Diesel use¶ | 0.06 | 0 | 0.06 | (0.06) | (1, 2) |
| Plant capital/equipment | 0.44 | 0.13 | 0.44 | 0.13 (0.31) | (2) |
| Process water | 0.29 | 0.29 | 0.29 | 0.29 | (2) |
| Sewage effluent | 0.29 | 0.29 | 0.29 | 0.29 | (2) |
| Pretreatment/Enzymes | 1.2 | 0.6 | 1.2 | 0.6 (1.2) | (6) |

* Overall, agricultural energy inputs from the continuous corn system were lower than in other studies (2, 7) because liming applications, phosphorous applications, potassium applications, insecticide applications, and irrigation energy requirements were not used, which collectively, contribute substantially to total agricultural energy requirements for corn production. Embodied nitrogen fertilizer energy values are for ammonium nitrate.

**Herbicides were applied in two out of the eight years (value shown was averaged across study period**)**.

*** Herbicide energy values were less than previous corn grain ethanol studies (2, 7) because glyphosate was the only herbicide used. Proportion of herbicide energy was allocated to stover based on grain:stover harvest ratio.

† Machinery and infrastructure requirements were variable by feedstock and harvest method. For no-till corn, machinery and infrastructure requirements are: no-till planter, self-propelled herbicide sprayer, combine, grain cart with auger, fertilizer applicator (shank), grain auger, large size mechanical front wheel drive (MFWD) tractor (160 kW), medium size MFWD tractor (97 kW), and grain bins. We assume that the combine spreader is disengaged to windrow the stover. Corn stover is assumed to be harvested separately from corn grain harvest. Machinery requirements for corn stover harvest are a baler, pull-type bale handler, small size MFWD tractor (71 kW), and large size MFWD tractor (142 kW). Switchgrass machinery requirements for the establishment year are: no-till drill, self-propelled herbicide sprayer, self-propelled mower/conditioner, and baler (assume 50% of bale operation is from a large, rectangular baler and 50% a large, round baler), pull-type bale handler, small size MFWD tractor (71 kW), and large size MFWD tractor (142 kW). Post-establishment switchgrass machinery requirements are: fertilizer cart, self-propelled boom sprayer, self-propelled mower/conditioner, baler, pull-type bale handler, small size MFWD tractor (71 kW), and large size MFWD tractor (142 kW). The analysis excluded the fertilizer cart in switchgrass treatments that received 0 kg N ha^-1^ and included the self-propelled boom sprayer in years where herbicides were applied (2 out of the 8 growing seasons for switchgrass).

‡ Biomass production system diesel use was estimated based on the number and type of field operations in a given year. Agricultural diesel estimates are based on field capacity to seed, grow, and harvest the respective feedstocks. Farm diesel use was 595 MJ ha^-1^, 1308 MJ ha^-1^, and 1361 MJ ha^-1^ for the 0 kg N ha^-1^, 60 kg N ha^-1^, and 120 kg N ha^-1^ switchgrass treatments, respectively. Switchgrass diesel estimates are based on previous analysis from field-scale harvests (8). Harvest diesel estimates are allocated separately to the corn grain harvest phase and stover removal phase. Diesel requirements for corn are based on previous no-till estimates (5). Diesel requirements for corn production were lower than previous estimates through the use of no-till practices.

¶Diesel use for cellulosic biomass transport at a cellulosic biorefinery.

Table S3. Petroleum requirements for the conversion phase of a natural gas (NG) dry mill (corn grain), co-located ethanol plant (corn grain and stover) and a separate cellulosic (corn stover or switchgrass) ethanol plant.

|  | NG dry mill | Co-located | Cellulosic |
| --- | --- | --- | --- |
|  | ───MJ petroleum L^-1^ ethanol─── | | |
| Feedstock transport | 0.59 | 0.60 | 0.63 |
| Plant capital | 0.05 | 0.18 | 0.18 |
| Electricity | 0.02 | 0.02 | 0.00 |
| Wastewater and treatment | 0.24 | 0.24 | 0.24 |
| Chemical/enzymes | 0.24 | 0.30 | 0.48 |
| Co-product credit | -0.71 | -0.71 | -0.12 |

Table S4. Greenhouse gas emission values for the agricultural phase from corn grain, corn grain and stover, and switchgrass. Values from direct soil C were derived from Follett et al. (9).

| Cropping System | N Rate | Fossil Fuel | Direct Soil C | Fertilizer* | TOTAL |
| --- | --- | --- | --- | --- | --- |
|  | ─kg N ha^-1^ yr^-1^─ | ─────────kg CO_2_e ha^-1^ yr^-1^───────── | | | |
| Corn grain | 60 | 397 | -8827 | 786 | -7644 |
|  | 120 | 490 | -8592 | 1421 | -6681 |
|  | 180 | 386 | -9777 | 2021 | -7370 |
|  |  |  |  |  |  |
| Corn grain and stover | 60 | 441 | -7599 | 690 | -6468 |
|  | 120 | 428 | -6990 | 1333 | -5229 |
|  | 180 | 432 | -9716 | 1952 | -7332 |
|  |  |  |  |  |  |
| Switchgrass (August) | 0 | 98 | -4892 | 0 | -4794 |
|  | 60 | 153 | -9032 | 746 | -8133 |
|  | 120 | 179 | -7201 | 1355 | -5667 |
|  |  |  |  |  |  |
| Switchgrass (Killing Frost) | 0 | 118 | -5401 | 0 | -5283 |
|  | 60 | 181 | -7255 | 985 | -6089 |
|  | 120 | 204 | -7133 | 1658 | -5271 |

*Fertilizer GHG emission values are the combination of nitrogen production values and N_2_O emissions (direct and indirect) from nitrogen fertilizer application. For direct N_2_O emissions it was assumed that 1% of applied N and 1% of the N contained in aboveground biomass retained in the field was lost as N_2_O. Corn root biomass was assumed to be 22% of aboveground biomass while switchgrass root biomass was assumed to be 33% of aboveground biomass. Nitrogen concentrations in aboveground biomass and belowground roots were derived from the BESS model (4). The amount of aboveground biomass left in the field differed by crop and whether stover was removed. For corn with stover removal, the amount of aboveground biomass left in the field was determined as the amount of corn grain harvested minus corn stover harvested. For switchgrass, a constant harvest height was used in all treatments so aboveground biomass retained was the same across all treatments. For switchgrass, a biomass value of 1.5 Mg ha^-1^ was estimated to be retained on the field. Indirect emission from switchgrass and corn were estimated from volatilization and leaching/runoff potential. It was assumed that 10% of the applied fertilizer was lost through volatilization and 1% of this quantity is denitrified to N_2_O. For leaching/runoff, we assumed that 30% of the applied N is lost from leaching/runoff and 0.75% of this amount is denitrified (4). We are likely overestimating indirect N_2_O emissions from the switchgrass treatments based on previous results which indicate lower N leaching and runoff potential from perennial grass systems than annual crops (10-13). Soil methane emissions were not accounted for as overall fluxes are small and are generally a minor sink for aerated cropland (14). We allocated a portion of soil C storage to the corn grain and stover based on amount harvested under the separate ethanol facility scenario. Since approximately 50% of stover biomass was harvested compared to grain, carbon savings from sequestration was proportionally allocated to stover based on the corn:stover harvest ratio for each replicated plot.

Table S5. Greenhouse gas emissions (g CO_2_ e L^-1^ ethanol) of switchgrass and corn from conversion, co-products, and distribution phase. Biofuel cropping system evaluated are corn grain-only harvest (Corn Grain) processed at a natural gas dry mill ethanol plant; corn grain with stover removal (Grain + Stover) processed at a natural gas dry mill (grain) and at a separate cellulosic ethanol plant (stover); corn grain with stover removal processed at a co-located grain and cellulosic ethanol plant; and switchgrass processed at a cellulosic ethanol plant.

| System | Conversion | Co-Products | Distribution | Source(s) |
| --- | --- | --- | --- | --- |
|  | ─────────g CO_2_ e L^-1^───────── | | |  |
| Corn Grain | 681 | -347 | 30 | (2, 4) |
| Grain + Stover | 681 (341)* | -347 (-304) | 30 | (1) |
| Co-located | 308 | -347 | 30 | (1) |
| Switchgrass | 341 | -304 | 30 | (1, 2) |

*Values in parenthesis are for the corn stover portion.

References

1. Greenhouse Gases, Regulated Emissions, and Energy Use in Transportation (GREET) (2012) Argonne National Laboratory <http://greet.es.anl.gov/>.

2. Renewable and Applicable Energy Laboratory (2007) Energy and resources group biofuel analysis meta-model <http://rael.berkeley.edu/sites/default/files/EBAMM/>.

3. Farrell AE*,* Plevin RJ, Turner BT, Jones AD, O’Hare M, et al. (2006) Ethanol can contribute to energy and environmental goals*.* Science 311: 506-508.

4. Liska AJ*,* Yang HS, Bremer VR, Erickson G, Klopfenstein T, et al. (2008) BESS: Biofuel energy systems simulator; life-cycle energy and emissions analysis model for corn-ethanol biofuel (v. 3).

5. Liska AJ*,* Yang HS, Bremer VR, Klopfenstein TJ, Walters DT, et al. (2009) Improvements in life cycle energy efficiency and greenhouse gas emissions of corn-ethanol*.* J Ind Ecol 13(1): 58-74.

6. MacLean HL, Spatari S (2009) The contribution of enzymes and process chemicals to the life cycle of ethanol*.* Environ Res Letters 4(1): 014001.

7. Hill J, Nelson E, Tilman D, Polasky S, Tiffany D (2006) Environmental, economic, and energetic costs and benefits of biodiesel and ethanol biofuels*.* Proc Natl Acad Sci USA 103(30): 11206-11210.

8. Schmer MR, Vogel KP, Mitchell RB, Perrin RK (2008) Net energy of cellulosic ethanol from switchgrass*.* Proc Natl Acad Sci USA 105(2): 464-469.

9. Follett RF, Vogel KP, Varvel GE, Mitchell RB, Kimble J (2012) Soil carbon sequestration by switchgrass and no-till maize grown for bioenergy*.* BioEnergy Res 5: 866-875.

10. McIsaac GF, David MB, Mitchell CA (2010) Miscanthus and switchgrass production in central illinois: Impacts on hydrology and inorganic nitrogen leaching*.* J Environ Qual 39(5): 1790-1799.

11. McLaughlin SB*,* et al. (2002) High-value renewable energy from prairie grasses. Environ Sci Technol 36: 2122-2129.

12. Randall GW, Mulla DJ (2001) Nitrate nitrogen in surface waters as influenced by climatic conditions and agricultural practices*.* J Environ Qual 30: 337-344.

13. Wilson HM, Cruse RM, Burras CL (2011) Perennial grass management impacts on runoff and sediment export from vegetated channels in pulse flow runoff events. Biomass Bioenergy 35: 429-436.

14. Schmer MR, Liebig MA, Hendrickson JR, Tanaka DL, Phillips RL (2012) Growing season greenhouse gas flux from switchgrass in the northern great plains*.* Biomass Bioenergy 45: 315-319.
